# Automated audio recording as a means of surveying tinamous (Tinamidae) in the Peruvian Amazon

**DOI:** 10.1002/ece3.8078

**Published:** 2021-09-03

**Authors:** Reid B. Rumelt, Arianna Basto, Carla Mere Roncal

**Affiliations:** ^1^ College of Agriculture and Life Sciences Cornell University Ithaca NY USA; ^2^ Warner College of Natural Resources Colorado State University Fort Collins CO USA; ^3^ School of Forest, Fisheries, and Geomatics Sciences University of Florida Gainesville FL USA

**Keywords:** bioacoustics, bird biology, machine learning, Neotropics, Peru, tinamous

## Abstract

1. The use of machine learning technologies to process large quantities of remotely collected audio data is a powerful emerging research tool in ecology and conservation.
2. We applied these methods to a field study of tinamou (Tinamidae) biology in Madre de Dios, Peru, a region expected to have high levels of interspecies competition and niche partitioning as a result of high tinamou alpha diversity. We used autonomous recording units to gather environmental audio over a period of several months at lowland rainforest sites in the Los Amigos Conservation Concession and developed a Convolutional Neural Network‐based data processing pipeline to detect tinamou vocalizations in the dataset.
3. The classified acoustic event data are comparable to similar metrics derived from an ongoing camera trapping survey at the same site, and it should be possible to combine the two datasets for future explorations of the target species' niche space parameters.
4. Here, we provide an overview of the methodology used in the data collection and processing pipeline, offer general suggestions for processing large amounts of environmental audio data, and demonstrate how data collected in this manner can be used to answer questions about bird biology.

## Graphical abstract

The use of machine learning technologies to process large quantities of remotely collected audio data is a powerful emerging research tool in ecology and conservation. We used autonomous recording units to gather environmental audio over a period of several months at lowland rainforest sites in the southeastern Peruvian Amazon and developed a Convolutional Neural Network‐based data processing pipeline to detect tinamou (Tinamidae) vocalizations. The resulting occurrence dataset is comparable to similar metrics derived from an ongoing camera trapping survey at the same site, and it should be possible to combine the two datasets for future explorations of the target species' niche space parameters.

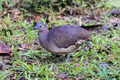

## 1 INTRODUCTION

Recent reductions in the size and cost of autonomous data collection equipment have allowed ecologists to better and more efficiently survey their study sites (Acevedo & Villanueva‐Rivera, 2006). Much work has been done to examine the benefits of using camera trap networks to detect shy and retiring species whose detection probabilities greatly decrease in the presence of human researchers (O'Connell et al., 2010). However, many species for which remote surveying techniques are optimal are difficult to properly monitor with camera traps due to their small body sizes and/or preference for heavy vegetative cover (Newey et al., 2015). A number of these species, particularly interior forest birds, are much easier to detect via acoustic monitoring techniques due to their frequent, far‐carrying vocalizations, and battery‐operated automated recording units (ARUs) have recently become a cost‐effective option for researchers working with these species (Brandes, 2008). ARUs can operate in the field for much longer periods of time than humans observers can (several days or weeks in many cases), efficiently and safely survey remote areas early in the morning and late at night, and, like camera traps, minimize disturbance to sensitive species. An additional benefit of audio recorders relative to camera traps is that audio recorders have a wider range of detectability than camera traps since they do not require direct line of sight, which increases the area of coverage and the likelihood of detecting rare species. However, in order to use audio recordings from ARUs, vocalizations from the target species must be detected among large quantities of survey audio. Efficiently and reliably identifying these detections presents a major challenge when developing a data processing pipeline. Although this task is still a nontrivial consideration in developing a study design, recent advancements in machine learning (ML) classification techniques, coupled with dramatic increases in the availability and accessibility of powerful hardware, have made this process easier than ever (Kahl et al., 2019). We strongly believe that the application of machine learning techniques to the processing of large quantities of automated acoustic event detection data will prove to be a transformative development in the fields of ecology and conservation, allowing researchers to tackle biological questions that have previously been impractical to answer.

**FIGURE 1:**
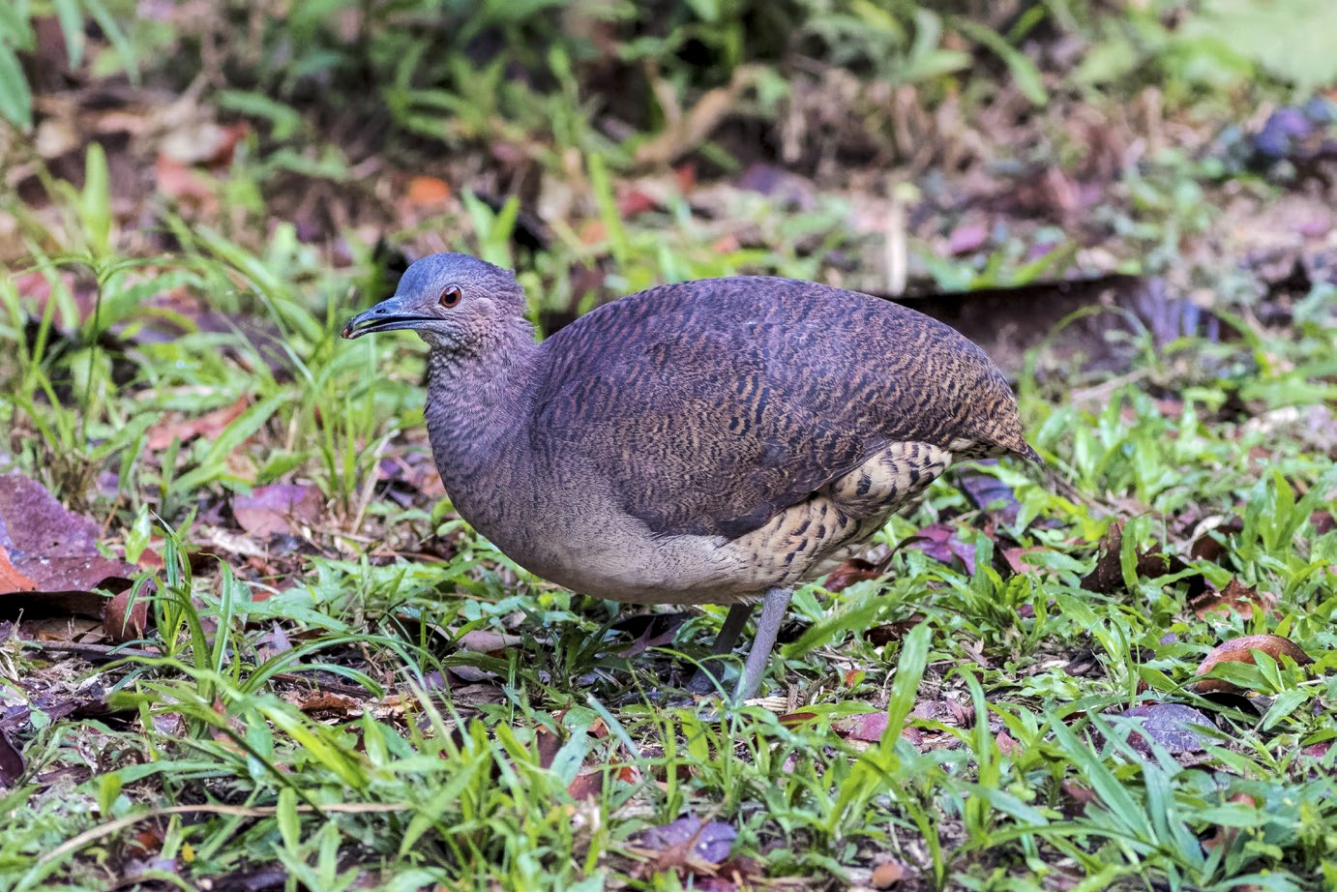
Undulated Tinamou (*Crypturellus undulatus*), a common floodplain tinamou species at LACC. Photo credit Reid Rumelt

Several life‐history characteristics of tinamous (Tinamidae; Figure 1), a group of terrestrial birds that occur widely in the Neotropics, make them superb candidates for field‐testing this type of audio processing pipeline. Although a few species in this family occupy open habitats, most show a high affinity for interior forest areas with thick vegetative cover (Bertelli & Tubaro, 2002). They are far more often heard than seen, and some species vocalize prolifically as part of the dawn and dusk choruses (Pérez‐Granados et al., 2020). This preference for interior forest, along with their large body sizes and terrestrial nature, makes tinamous inordinately susceptible to the effects of anthropogenic habitat change, in terms of both outright habitat loss and to increased human hunting pressure in fragmented forest patches near‐populated areas (Thornton et al., 2012). Intensive life‐history research in the coming years will be critical to conservation of tinamous and their habitats, and autonomous recording has the potential to revolutionize this line of inquiry.

Here, we present the preliminary results of an ongoing field study that involves deploying ARUs at lowland Amazonian forest sites in Madre de Dios, Peru. Although this region has tentatively among the highest levels of tinamou alpha diversity in the Neotropics (11 co‐occurring species: eBird, 2017), there is currently a lack of research into which biological and ecological factors allow such high degrees of alpha diversity. We collected environmental audio of each day's dawn and dusk choruses and designed a data pipeline that uses a machine learning (ML) audio classifier to identify tinamou vocalization events in the audio data and organize the detections into a spatiotemporal database for future use in producing occupancy models for the target species. To our knowledge, this technology has not previously been used to conduct community‐level surveying for tinamous and represents a promising alternative to camera traps and more traditional point‐count surveying as a means of studying elusive yet highly vocal bird taxa.

## 2 MATERIALS AND METHODS

### 2.1 Data collection

Data collection was conducted under the auspices of the Amazon Conservation Association at the Los Amigos Conservation Concession (LACC), in the lowland rainforest of Madre de Dios, Peru. This site, which protects ~145,000 ha of forest along the Río Los Amigos basin, is one of the most biodiverse lowland rainforest sites in the Amazon basin with close to 600 bird species, eleven of which are tinamous in the genera *Tinamus* and *Crypturellus* (eBird, 2017; Table 1). The station's biological diversity is due in part to its diversity of terrestrial microhabitats, which include terra firme and floodplain primary forest, secondary and edge forest, Guadua bamboo stands, and Mauritia flexuosa palm swamps (Larsen et al., 2006). As studies at this site (Mere Roncal et al., 2019) and elsewhere in the Neotropics have demonstrated that tinamou species differ in their specific habitat utilization characteristics (Guerta & Cintra, 2014), LACC is an exemplary site for detecting tinamous across a variety of habitat gradients.

**TABLE 1:** List of tinamou species at Los Amigos Biological Station, with source audio totals (first classification totals/second classification totals)

Common name	Scientific name	Training audio	Validation audio
Gray Tinamou	*Tinamus tao*	370/419	50/50
Great Tinamou	*Tinamus major*	252/2,000	50/50
White‐throated Tinamou	*Tinamus guttatus*	263/1,266	50/50
Cinereous Tinamou	*Crypturellus cinereus*	252/2,000	50/50
Little Tinamou	*Crypturellus soui*	276/461	50/50
Undulated Tinamou	*Crypturellus undulatus*	311/2,000	50/50
Brown Tinamou	*Crypturellus obsoletus*	255/255	50/50
Brazilian Tinamou	*Crypturellus strigulosus*	242/2,000	50/50
Black‐capped Tinamou	*Crypturellus atrocapillus*	79/79	25/25
Variegated Tinamou	*Crypturellus variegatus*	200/2,000	50/50
Bartlett's Tinamou	*Crypturellus bartletti*	320/2,000	44/50
Nontinamous audio	—	294/4,000	750/750

**FIGURE 2:**
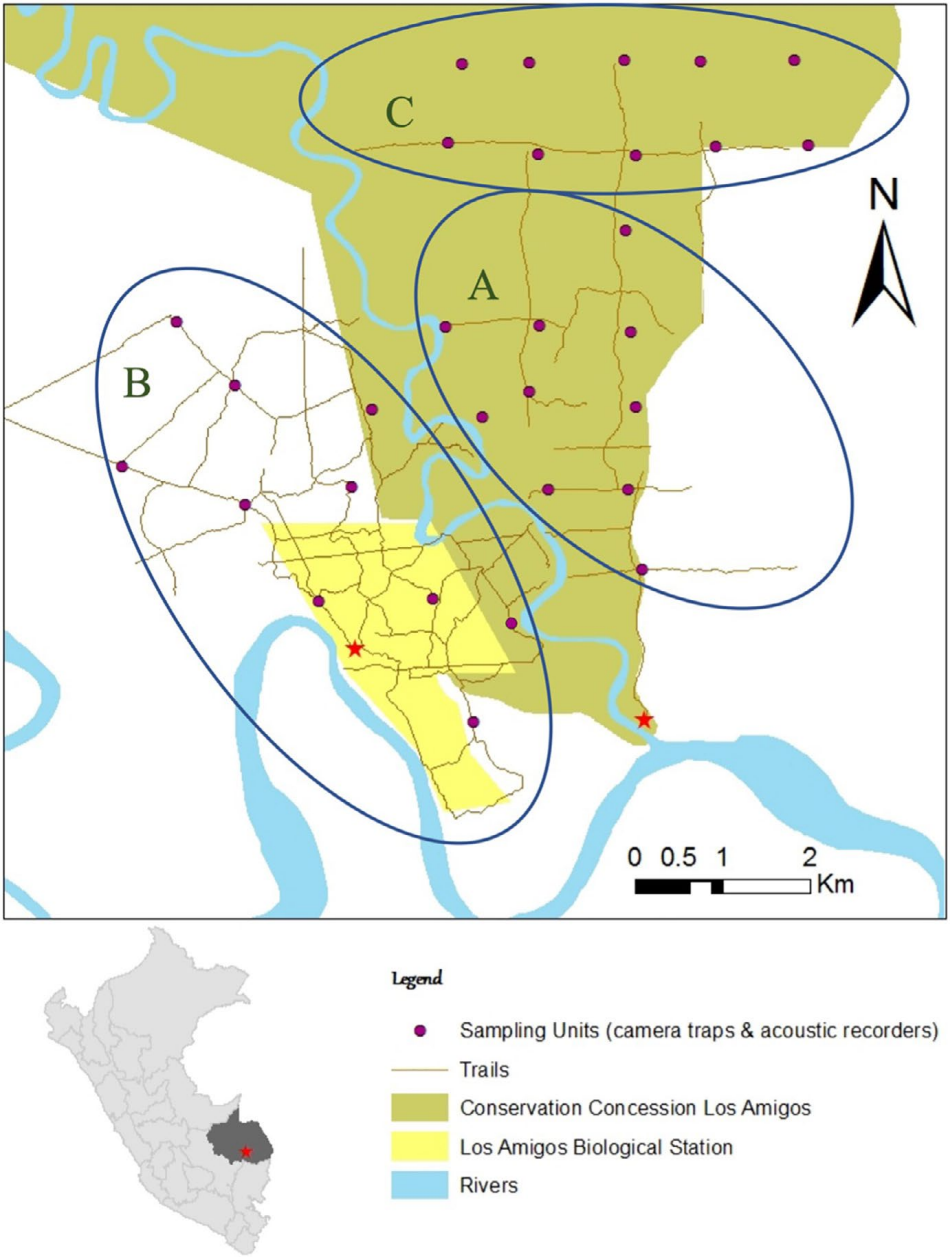
Map of survey points (subset of Los Amigos camera trap network). Group A: deployment 1; group B: deployments 2 & 4; group C: deployment 3

**FIGURE 3:**
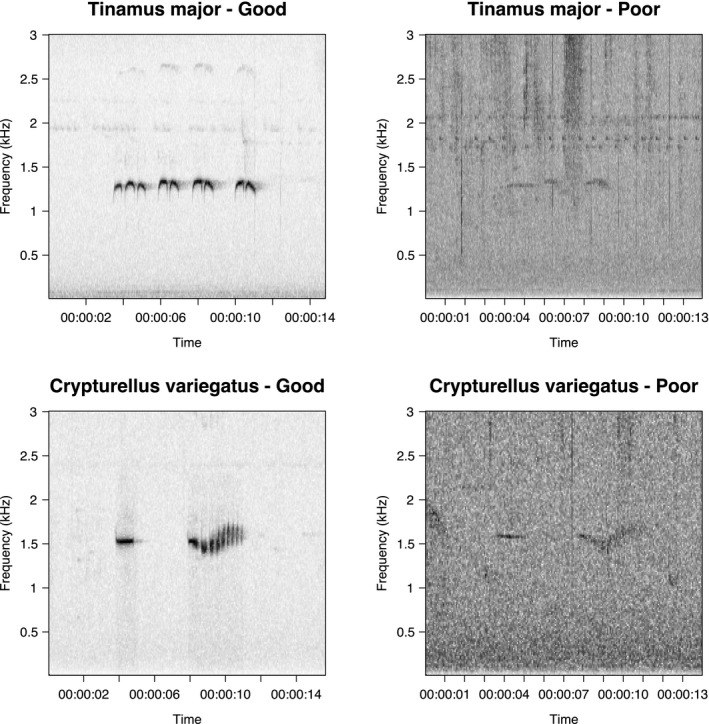
Example vocalizations of two common tinamou species at LACC, Great Tinamou (*Tinamus major*) and Variegated Tinamou (*Crypturellus variegatus*), collected from survey audio. Spectrograms vary drastically in quality depending on ambient noise and distance to recorder

Acoustic monitoring was conducted using ten SWIFT ARUs (Kahl et al., 2019), provided by the Cornell Lab of Ornithology, from mid‐July to early October of 2019. This period overlaps with the latter half of the dry season at LACC. The SWIFT units were deployed on rotating 14‐day deployment periods at terra firme and floodplain forest sites (Figures 2 and S1), 10 sites at a time, over three deployments from mid‐July to late August. A fourth deployment, duration 27 days, was conducted as a follow‐up at five of the 30 sites from late September to early October. As the chosen sites are part of the station's existing camera trap system (approximately a 1‐km^2^ grid located along the edge of open trails), we were able to merge our detection set with previously collected site‐level habitat data as well as to compare our tinamou detection rates to those calculated using camera trap detections. Recorders were tied to trees at a height of approximately 1.5 m from the ground with the microphone facing downwards. Each unit was programmed to record for five hours a day, from 5:00 to 7:30 in the morning and 16:00 to 18:30 in the afternoon to early evening, in order to cover periods of high vocal activity for tinamous (Dias et al., 2016). The SWIFT unit firmware allows for control of microphone gain and sampling frequency; we set these values to −33 dB (the default) and 16 kHz, respectively. Setting the sampling frequency to 16 kHz is a trade‐off that limits the acoustic frequency bandwidth to 0–8 kHz (Landau, 1967) in exchange for smaller file sizes and lower power demands than the default value of 32 kHz. These recording parameters are entirely acceptable for capturing the vocalizations of tinamou species at this site, all of which have vocalizations that are 900–2900 kHz (Figure 3). The SWIFT firmware writes data as 30‐min‐long WAV files (~58 MB). Each unit was intended to collect data for the shorter of (a) the entire 14‐ or 27‐day recording period or (b) until battery power was exhausted. In practice, battery life was always the limiting factor, with a mean time‐to‐shutdown of 7.81 days (5.12 days for deployments 1–3 and 21.8 days for deployment 4). Due to supply limitations, we were forced to use a different brand of battery for deployments 1–3 than for deployment 4, which we suspect is at least partially responsible for the longer per‐recorder run times in the latter deployment. At the end of each deployment period, all units were removed from the field, loaded with fresh recording media and batteries, and deployed to their next assigned site on the following day. All audio data were backed up to rugged solid‐state storage media for transport out of the field.

Our chosen classification procedure is a type of supervised machine learning, which requires a significant amount of training audio to produce a working model (Kotsiantis et al., 2007). We used a set of ~3,100 audio files of 2‐s duration (the typical phrase length in tinamou calls) to train an initial classifier. These files were coded as one of twelve classes: one class for each tinamou species and a “junk” class containing audio of other bird species, nonbird organismal audio, and assorted environmental audio (Table 1). The training dataset was derived from audio downloaded from the Macaulay Library of Natural Sounds (https://macaulaylibrary.org) and Xeno‐Canto (http://www.xeno‐canto.org) databases (S2) as well as from exemplar cuts in the audio we collected in the field. Effort was taken to ensure that the training examples covered the full breadth of the acoustic parameter space of tinamous, including for the two species in this study, *Crypturellus soui* and *Crypturellus variegatus*, that were observed to use distinct call and song types in the survey audio. All audio was checked to ensure correct assignment to species before use.

### 2.2 Data processing and classification

A series of preprocessing steps were applied to the audio after collection, beginning with normalizing all survey audio to −2 dB maximum gain. The SWIFT recorder firmware writes a high amplitude audio spike at the beginning of the first file recorded after the unit wakes from standby (e.g., the beginning of the 5:00 and 16:00 audio files); therefore, we chose to overwrite the first five seconds of audio on each of these files to prevent this spike from impacting the gain normalization step. As our chosen audio classifier architecture operates on fixed‐length samples, we split each 30‐min audio file into 7,197 2‐s‐long overlapping audio “windows” that advance forward by 0.25 s per window. The classifier operates on a section of the log‐Mel‐weighted spectrogram (Knight et al., 2017) of each window between 900 and 2,900 Hz, which is created dynamically during classification using STFT utilities in the TensorFlow python module (Table 2) at a native resolution of 512 × 512 px.

**TABLE 2:** STFT settings

Setting	Value
Dimensions	512 × 512 px
Channels	1 (grayscale)
Window size	1,024
Stride	64
Frequency band	900–2,900 Hz
Sampling rate	16 kHz
Mel bin number	64

Audio event detection was conducted using a set of Convolutional Neural Network classifiers. The chosen classifier architecture is adapted from the multiclass single‐label classifier called “Model 1” in Kahl et al. (2017). Our decision to use a multiclass single‐label classifier architecture was driven by a desire for reduced learning complexity; however, we feel there is merit to introducing a multilabel classifier in future analyses as existing ML techniques are capable of dealing with this task with minor modifications (Kahl et al., 2017). For similar reasons, we reduced the number of neurons per hidden layer by half to account for limitations in available processing power and also down‐sampled the 512 × 512 px spectrogram images to 256 × 256 px before training and classification. The full classifier architecture is described in Table 3. All data processing was performed either in Python, using a combination of TensorFlow 2.0 (Abadi et al., 2016) and other widely used Python modules, or, in the case of later statistical testing, in R (R Core Team, 2019). During training, we applied the same STFT algorithm as used for the survey data to dynamically convert the training audio to log‐Mel‐weighted spectrograms, and implemented data augmentation to improve model generalization (Ding et al., 2016). These augmentation parameters, along with general model hyperparameters (Table 4), were chosen using a Bayesian hyperparameter search module in Python (Nogueira, 2014) that was driven to optimize the calculated multiclass F1‐score (Sokolova & Lapalme, 2009) (*β* = 1) on a set of known good clips (hereafter the “validation set”) created using a sample of clips not used in the training data (Table 1). F1‐score was calculated as a macro‐average of the 12 classes in order to give equal weight to rare classes. Although the goal of hyperparameter search techniques is typically to identify an optimal set of parameters, we observed two apparent local optima that we chose to incorporate into our classification pipeline as two submodels: (a) submodel 1, which added artificial Gaussian noise to training spectrograms as part of the augmentation process and (b) submodel 2, which did not. The set of class probabilities returned for each clip was the mean of the probabilities reported by the two models (hereafter the “ensemble”). We validated each submodel, as well as the ensemble, on the same validation set used in hyperparameter search.

**TABLE 3:** CNN architecture

Layer type	Details
Input	Size 256 × 256 × 1
Conv2D	Size 32 × 7 × 7, Stride 2
MaxPooling2D	Size 2
Conv2D	Size 64 × 5 × 5, Stride 1
MaxPooling2D	Size 2
Conv2D	Size 128 × 3 × 3, Stride 1
MaxPooling2D	Size 2
Conv2D	Size 256 × 3 × 3, Stride 1
MaxPooling2D	Size 2
Conv2D	Size 512 × 3 × 3, Stride 1
Flatten	(None)
Dense	256 Units
Dropout	0.5
Dense	4 Units

Note

L2 kernel and activity regularization (1e−06, with default biases turned off) were applied to each Conv2D layer, with batch normalization (momentum = 0.01) applied between the Conv2D and MaxPooling2D layers. ReLU activation was used for all Conv2D layers and the first Dense layer, with Softmax activation applied to the output layer.

**TABLE 4:** Ranges and chosen values for hyperparameters and augmentation parameters

Parameter	Type	Range	Value
Batch size	Hyperparameter	[16, 32, 64, 128]	64
Dropout	Hyperparameter	[0.2, 0.5]	0.5
Epochs	Hyperparameter	20	20
L2 amount	Hyperparameter	[1e−6, 1e−2]	1e−6
Learning rate	Hyperparameter	[1e−9, 1e−2]	0.0075
Network size scale	Hyperparameter	[1, 2, 3, 4]	2
Gaussian noise intensity	Augmentation	[0, 20]	0.8
Gaussian blur	Augmentation	[0, 3]	0
Horizontal shift	Augmentation	[0, 50 px]	[0, 20 px]
Random dB offset	Augmentation	[0, −40 dB]	[0, −40 dB]
Vertical shift	Augmentation	[0, 5 px]	[0, 2 px]

Note

Single value ranges indicate that the parameter was held constant. Zero‐values for continuous parameters indicate that the parameter was not used. Values that are ranges indicate that values were randomly chosen from this range on a per‐spectrogram basis (augmentation only).

When deployed on survey data, our classification pipeline yields classifications as a sequence of probability vectors of size 12, where each vector corresponds to one window in the sequence of overlapping windows. Raw class probabilities for windows that contain only the very beginning or the very end of tinamou vocalizations are often classified incorrectly, which we believe results from the fact that different tinamou species often share structural similarities with one another in those regions of their vocalizations. To reduce the impact of this pattern on our overall classification accuracy, we applied a “smoothing” postprocessing to the class probabilities where each probability value was replaced by the weighted average of that value (weight = 1) and the values immediately before and after it in the time sequence (weight = 0.5). Windows with a maximum class probability < 0.85 were removed, and the remainder assigned the label with the highest class probability. All windows detected as positive were manually checked for accuracy and relabeled if incorrect.

We assessed the degree of marginal improvement in classifier performance due to increased training dataset size and increased structural uniformity between training clips and survey audio by running a second “pass” of the acoustic classifier on the survey data with a set of models that had been trained using a larger training dataset. To generate this dataset, the original training dataset was supplemented with all ground‐truthed positive windows from the initial classification (the first “pass”). We sampled from this dataset to produce a new training dataset (*n* = 18,480) with the larger of 2,000 randomly selected clips (4,000 for the “junk” class), or as many clips as were available, per class (Table 1). We trained new submodels on these data using the same model architecture and hyperparameters that were used for models in the first pass. The sole change made to the training process between classifications 1 and 2 was to alter the batch generation code to produce batches with balanced class frequencies to offset the greatly increased degree of class imbalance in the supplemented dataset. Each submodel was validated using a new validation set that contained known good survey audio whenever possible in order to ensure that the calculated metrics would be more indicative of each submodel's real‐world performance.

The survey data were classified with these new models, and the resulting class predictions were processed to extract probable detections as described previously. In order to decrease labor time, all positive windows from the initial classification were “grandfathered in” as correctly identified due to having been manually checked previously, which allowed us to only check positive detections that were newly identified during the second pass. Finally, all sequences of windows with a particular species classification that were ≥0.75 s apart from any other sequence were grouped as a single vocal event.

For the purposes of quantifying model performance and generalizability, we calculated a precision, recall, F1‐score, and precision–recall area under the curve (AUC) performance metrics for the primary and secondary models, presented on a per‐class basis or as macro‐averages across classes, after Sokolova and Lapalme (2009). All metrics were calculated based on classifier performance on a set of known good clips (hereafter the “validation set”), using data from the survey audio whenever possible in order to ensure that the performance metrics would be more indicative of each submodel's real‐world performance.

When applying a novel survey methodology to a system, it is important to establish a baseline with which to compare survey results. We chose to compare our audio detection counts to camera trap capture rates for tinamous reported by Mere Roncal et al. (2019), which was conducted from February 2017 to June 2018 at LACC. Camera trap capture rates suggest seasonally driven differences in tinamou activity rates, so we only considered detection rates from the dry season portion of this study (July–September 2017), which limited our comparison to the five tinamou species reported by Mere Roncal et al. (2019) for which dry season camera trap data are available. This study reports frequencies as captures per 1,000 hr of recording time, which allows us to correct for site‐level differences in survey effort (3 months per site for camera traps vs. 2 weeks for acoustic monitors). Due to the relatively small number of camera trap captures per site, we chose to examine aggregate values rather than comparing recorder and camera trap data from each site. Following Mere Roncal et al. (2019), we used independent captures as the basis for our comparison, which involves lumping detections separated by less than one hour. Occurrence frequencies were calculated from the acoustic monitoring data as the average of the distributions from terra firme and floodplain sites. In addition, we compared detection effort and cost per unit detection between the two survey methods by comparing capture rates to equipment costs from the two studies (S4). Detection effort multiplier was calculated as the ratio of capture rates, and cost per unit survey effort was calculated as total equipment cost divided by capture rate.

We also examined community science observation data for tinamous from eBird, as this dataset represents a potential source of data to use in future species distribution modeling studies in this region (Sullivan et al., 2009, 2014). We used stationary and traveling checklists containing tinamous that were submitted at the LACC hot spot between the months of July and October, removing stationary checklists with durations >150 min and traveling checklists with lengths >0.5 km in order to constrain the sampling effort parameter space of the eBird data such that it was more comparable to our 2.5‐hr morning and afternoon recording periods. Despite these filtering steps, the final eBird dataset still contained all locally occurring tinamou species. However, it was clear that our acoustic data density for *Crypturellus strigulosus* vastly outstripped eBird data density, so we excluded this species from our analysis as we feel it warrants separate discussion. We produced estimates of occurrence probabilities from the filtered eBird dataset by sampling checklists containing tinamous and tabulating presence–absence values for each tinamou species. We then examined the density distribution of eBird checklist durations and created a sampling kernel that could select periods of time from the acoustic monitoring dataset with random start times and lengths that follow this same density distribution. We used this kernel to estimate occurrence frequencies separately for terra firme and floodplain habitat types, and these values were averaged.

## 3 RESULTS

### 3.1 Model performance

**FIGURE 4:**
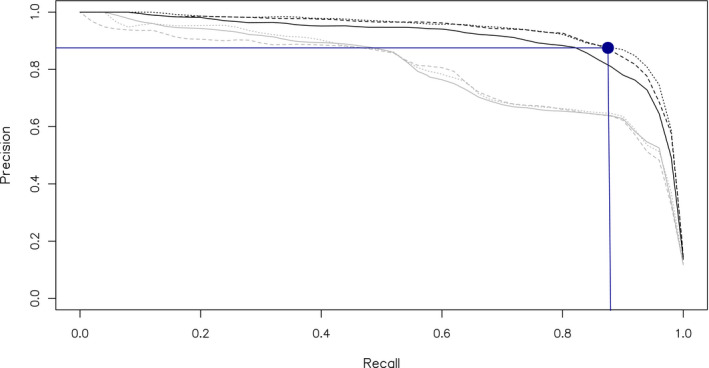
Precision–recall curves for submodels 1 (solid line), 2 (dashed line), and ensemble (dotted line) for classification passes 1 (gray) and 2 (black), macro‐averaged metrics. Dark blue lines indicate recall and precision measured at the chosen probability value for positive detections (*p* = 0.85)

**FIGURE 5:**
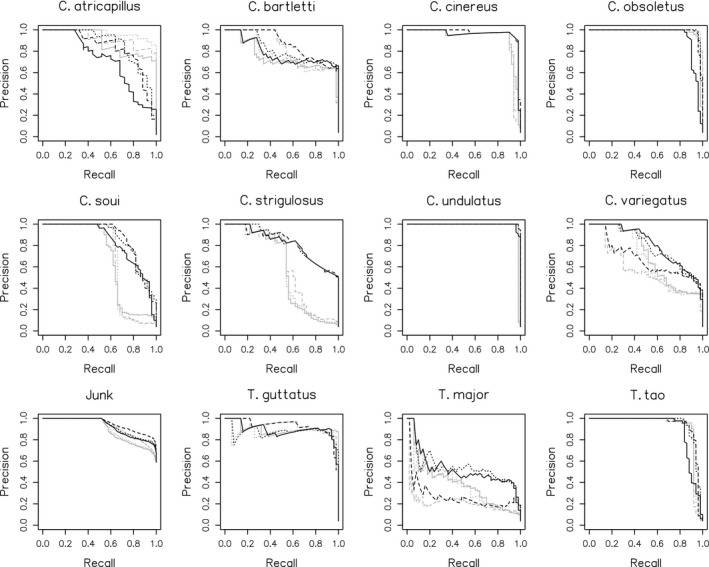
Precision–recall curves for submodels 1 (solid line), 2 (dashed line), and ensemble (dotted line) for classification passes 1 (gray) and 2 (black), per class

**FIGURE 6:**
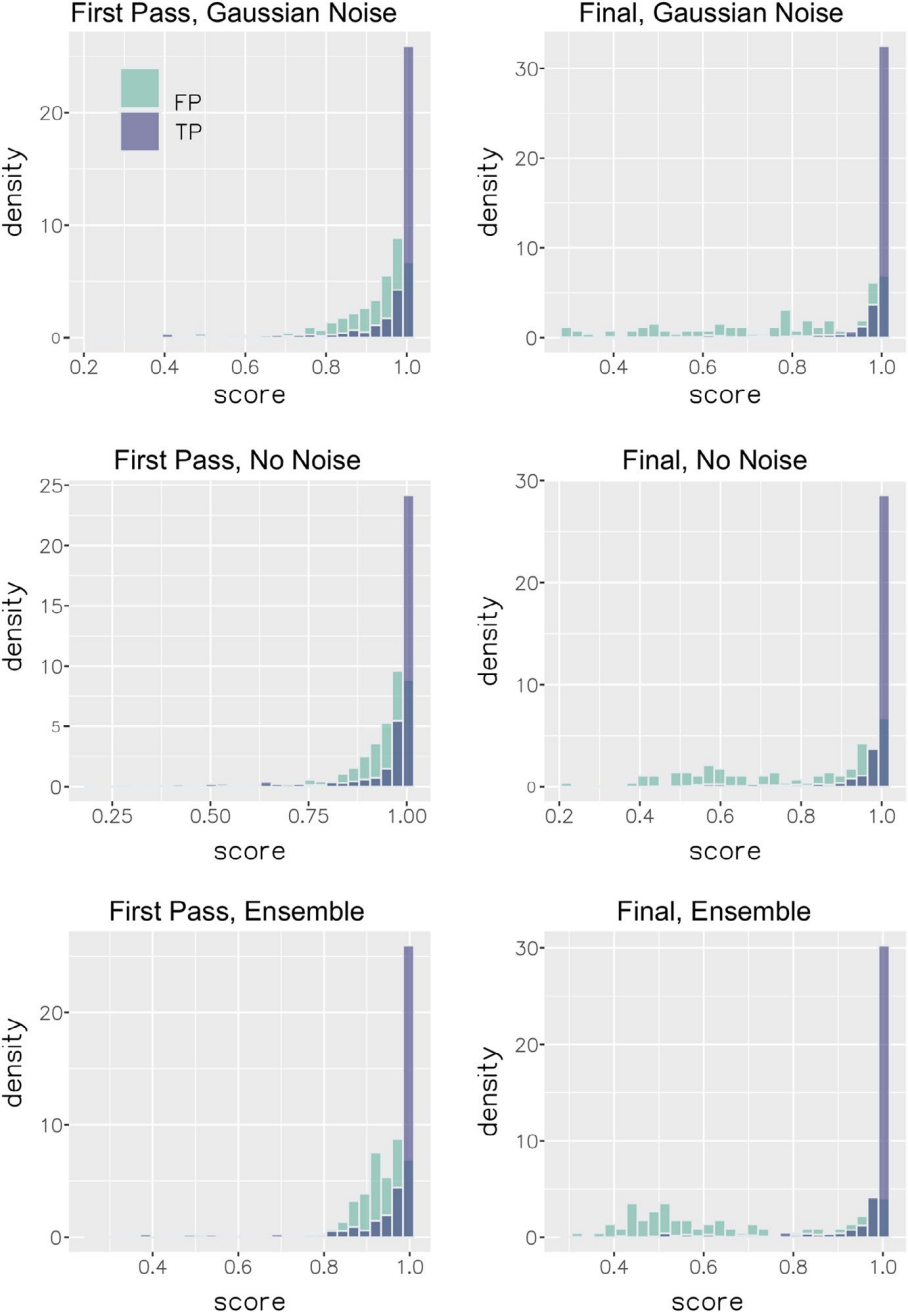
Relative density of classifier true‐positive (TP) and false‐positive (FP) detections on the validation set by submodel and training pass. “Final, Ensemble” (lower right) was the model used to classify survey data

The performance of all models is summarized in Figures 4, 5, 6 and Tables 5, 6. At the macro‐averaged level, the ensemble model performed better than either submodel individually within each classification pass (Table 5). The addition of random artificial noise in submodel 2 improved both precision and recall in pass 1, though only recall in pass 2 (Table 5). The ensemble model of pass 2 performed substantially better than the corresponding model in pass 1 (Figure 6), likely due to both the larger training dataset used for this pass and the fact that the training and validation datasets used during this pass were both comprised of audio collected by us in the field, thus being more similar to one another than they were in pass 1. This increased similarity between training and validation datasets in pass 2 is also a potential explanation for the observed decrease in recall score with added artificial noise during this pass, though we did not perform further analysis of this specific result. Per‐class performance was generally good, with visible improvements from pass 1 to pass 2 in most, though species with subjectively more variable vocalizations (e.g., *Tinamus major*) performed less well (Figure 5, Table 6). Intriguingly, the increase in classification accuracy we observed at the macro‐averaged level did not hold uniformly true at a class level, with submodel 1 or 2 often yielding better results (Table 6). An analysis of classifier score distributions for positive detections showed increased score separation between true‐positive and false‐positive detections in pass 2 relative to pass 1 (Figure 6), indicating better overall predictive power in the case of the latter model (Knight et al., 2017). We also observed that our chosen score threshold yielded precision and recall values that were close to the inflection point of the precision–recall curve, indicating this value was an appropriate choice for ensuring a good balance of the two metrics (Table 7).

**TABLE 5:** Overall classifier performance (after Knight et al., 2017; Sokolova & Lapalme, 2009)

Classification pass	Submodel	Precision	Recall	F1‐Score	prAUC
1	*No noise*	0.777	0.803	0.735	0.797
	*Artificial noise*	0.783	0.827	0.754	0.788
	*Ensemble*	0.803	0.845	0.773	0.803
2	*No noise*	0.878	0.846	0.820	0.909
	*Artificial noise*	0.853	0.890	0.856	0.933
	*Ensemble*	0.875	0.882	0.861	0.938

Abbreviation: prAUC, Precision–recall AUC.

**TABLE 6:** prAUC scores for each submodel type, per class

Classification pass	Species	No noise	Artificial Noise	Ensemble
1	*C. atricapillus*	0.891	0.921	0.968
	*C. bartletti*	0.748	0.826	0.770
	*C. cinereus*	0.934	0.930	0.926
	*C. obsoletus*	0.981	0.992	0.996
	*C. soui*	0.680	0.683	0.684
	*C. strigulosus*	0.577	0.614	0.597
	*C. undulatus*	0.982	0.981	0.981
	*C. variegatus*	0.703	0.576	0.671
	*Junk*	0.896	0.925	0.902
	*T. guttatus*	0.903	0.931	0.882
	*T. major*	0.386	0.216	0.387
	*T. tao*	0.933	0.915	0.932
2	*C. atricapillus*	0.536	0.705	0.725
	*C. bartletti*	0.995	0.964	0.994
	*C. cinereus*	0.981	0.984	0.982
	*C. obsoletus*	0.928	0.985	0.983
	*C. soui*	0.886	0.918	0.912
	*C. strigulosus*	0.965	0.960	0.965
	*C. undulatus*	0.996	0.999	0.999
	*C. variegatus*	0.919	0.939	0.938
	*Junk*	0.991	0.995	0.995
	*T. guttatus*	0.953	0.951	0.955
	*T. major*	0.913	0.899	0.920
	*T. tao*	0.892	0.944	0.936

Note

Filled‐in cells indicate the model with the maximum prAUC value for each class within the two passes.

**TABLE 7:** Confusion matrix for ensemble validation

	*C. atricapillus*	*C. bartletti*	*C. cinereus*	*C. obsoletus*	*C. soui*	*C. strigulosus*	*C. undulatus*	*C. variegatus*	*Junk*	*T. guttatus*	*T. major*	*T. tao*	Training Size
C. atricapillus*	6	0	0	2	4	0	0	2	1	7	0	3	79
*C. bartletti*	0	49	1	0	0	0	0	0	0	0	0	0	2,000
*C. cinereus*	0	1	48	0	0	0	0	0	1	0	0	0	2,000
C. obsoletus*	0	0	0	45	0	0	0	0	4	0	0	1	255
*C. soui*	1	0	0	0	41	0	0	0	1	4	0	3	461
*C. strigulosus*	0	0	0	0	0	50	0	0	0	0	0	0	2,000
*C. undulatus*	0	0	0	0	0	0	49	0	1	0	0	0	2,000
*C. variegatus*	0	0	0	0	0	0	0	47	2	0	0	1	2,000
*Junk*	0	3	0	0	3	8	1	12	707	2	12	2	4,000
*T. guttatus*	0	0	0	0	1	0	0	0	0	49	0	0	1,266
*T. major*	0	0	0	0	0	0	0	1	2	0	47	0	2,000
*T. tao*	0	0	0	0	0	1	0	0	2	2	0	45	419

Note

Columns are true species labels, rows are predicted species labels. Species with asterisks were ultimately not detected in the survey data.

Dark and light cell tones represent true and false detections for Crypturellus species (green), Tinamus species (blue), and junk audio (red).

### 3.2 Collection data and ecological analyses

**FIGURE 7:**
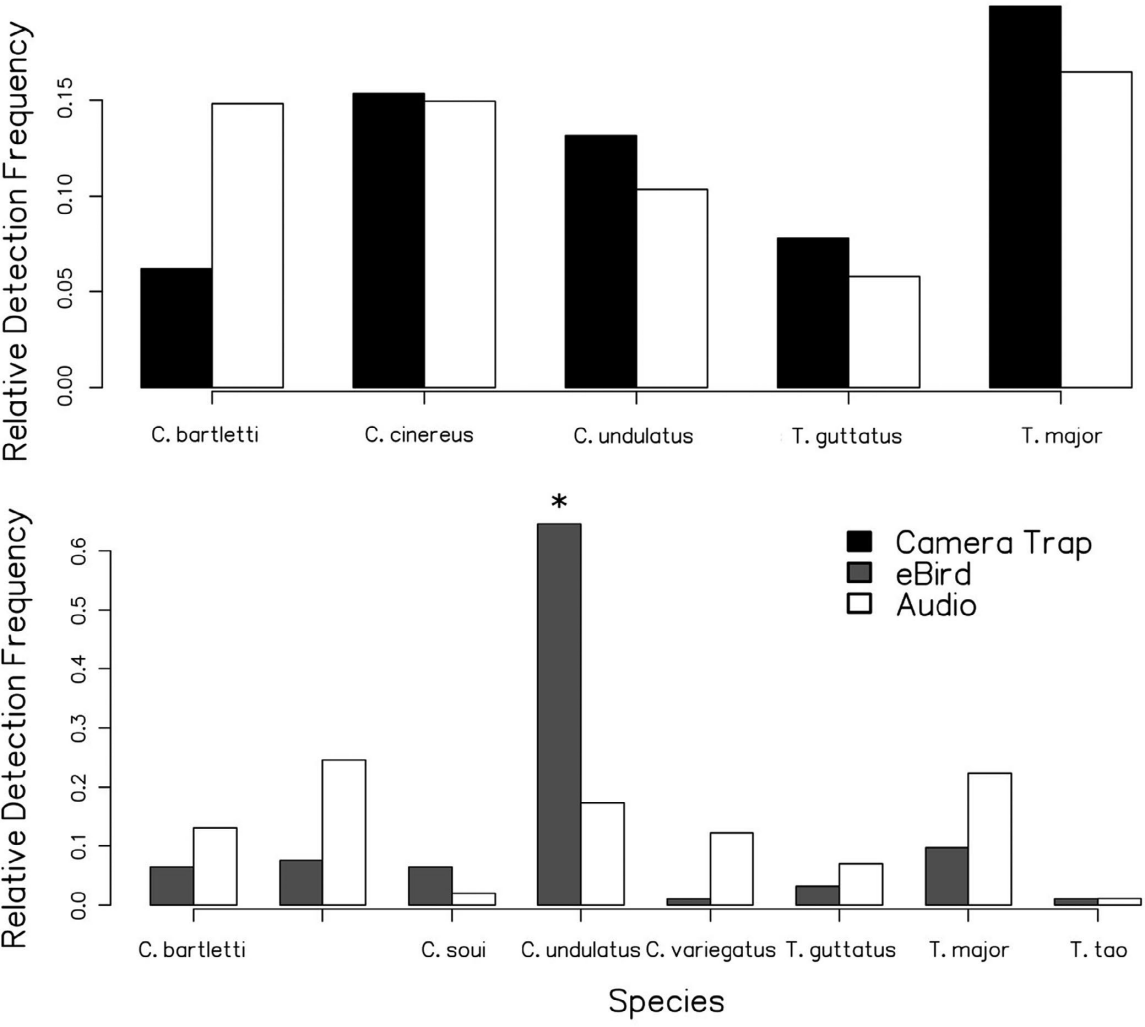
Relative audio event detection frequency versus relative detection frequencies from eBird and camera trap data

We collected a total of 1,216.5 hr of audio, of which 544.5 hs (45%) came from deployment 4 and 225.5 (19%), 201.0 (17%), and 245.5 hs (20%) came from deployments 1, 2, and 3, respectively (S3). The total number of recording hours per habitat type (899.0 hs in terra firme, 317.5 hs in floodplain) was roughly proportional to the number of site‐deployment combinations in each habitat type (24 vs. 13). We detected a total of 15,878 tinamou vocalization events, 2,189 of which were added after the second classification pass. Our detections represent nine of the 11 species present at LACC, with data densities ranging from 4,468 events for *C. strigulosus* to 26 for *Tinamus tao* (S3). Two species were not detected: *Crypturellus atricapillus* and *Crypturellus obsoletus*. Both species are uncommon at Los Amigos (eBird, 2017; personal obs.), are known to have affinities for brushy edge habitats that were located away from most of the recorders (Anjos, 2006; Cabot et al., 2020), and were entirely absent from the camera trap dataset. Therefore, we suspect that their lack of detection indicates true absence from the dataset rather than poor class performance. Removing nonindependent events yielded 771 captures (Table 8). The relative occurrence frequency of the tinamou species as measured by our audio detection pipeline differs significantly from the observation frequencies reported by eBird (*χ* ^2^ = 567.4, *p* < 2.2e−16; Figure 7b), but notably there was no significant difference between these frequencies and camera trap capture rates for the five species represented in both datasets (*χ* ^2^ = 0.072352, *p* > 0.1; Figure 7a). Acoustic monitoring was far more efficient as a survey method than camera trap surveying, collecting captures at a 301× higher rate and 147× lower cost per unit effort (Table 8).

**TABLE 8:** Analysis of detection effort and cost per detection for acoustic monitoring and camera trapping

	Acoustic captures	Acoustic capture rate	Cost Per acoustic capture per 1,000 trap days	Camera trap captures (estimated)	Camera trap capture rate	Cost per camera capture per 1,000 trap days
Bartlett's Tinamou	85	1,676.9	$1.67	0.029	3.1	$406.71
Cinereous Tinamou	131	2,584.5	$1.08	0.063	6.6	$191.03
Undulated Tinamou	58	1,144.3	$2.45	0.073	7.7	$163.74
White‐throated Tinamou	74	1,459.9	$1.92	0.037	3.9	$323.28
Great Tinamou	129	2,545	$1.10	0.095	10	$126.08
Total	**477**	**9,410.6**	**$8.22**	**0.297**	**31.3**	**$1,210.84**
Species not present in camera trap dry season dataset:						
Little Tinamou	11	217	$12.90	—	—	—
Brazilian Tinamou	119	2,347.7	$1.19	—	—	—
Variegated Tinamou	150	2,959.3	$0.95	—	—	—
Gray Tinamou	14	276.2	$10.13	—	—	—

Note

Camera trap captures are estimates made by applying the capture rates reported in Mere Roncal et al. (2019) to the survey duration of the acoustic monitoring project. In this system, the acoustic monitoring survey method collected detections at a 301× higher rate than camera trapping while being 147× less expensive per unit effort.

Bold values represent the sum for each column in the upper part of the table for which complete data is available.

## 4 DISCUSSION

The machine learning pipeline we used in this study appears to be an effective tool for collecting occurrence data across a range of habitat types at our target site, both in terms of data density and in terms of cost. Although the lack of statistically significant differences in relative detection frequencies between the audio and camera trap data conflicted slightly with our expectation that acoustic sampling would yield different occurrence metrics than camera trap sampling, it is encouraging that our frequencies are so similar as they may both be good approximations of the true values. Increasing the sample size of the camera trap dataset and collecting audio samples from the wet season may yet allow us to identify true underlying differences in detection probabilities for tinamous when surveyed acoustically versus visually.

The significant differences in detection frequency we observed between our data and the eBird data are likely a result of nonrandom spatial sampling. An example of this spatial nonrandomness with a clear causative explanation is the relatively higher eBird detection frequency for *Crypturellus undulatus*, a species that is present widely in floodplain and transitional forest but is also extremely common in edge habitat near the station dwellings where ecotourists and birders visiting the station spend time when not hiking on trails (eBird, 2017; personal obs). We chose not to include *C. strigulosus* in frequency analyses as it is represented in our audio dataset mainly by detections at sites east of the Río Los Amigos that birders and ecotourists visiting the station are rarely if ever able to access, therefore heavily limiting its sampling density in the eBird dataset (personal obs). However, even in the absence of quantitative assessment, we nonetheless believe this is another clear case of spatially nonrandom eBird sampling patterns relative to the more structured audio and camera trap data. We therefore advise caution when using eBird data to generate site‐level relative occurrence frequencies for tropical forest birds, as doing so properly requires a substantially better‐informed set of sample bias corrections than we chose to use for this illustratively naïve approach. eBird's own Status and Trends methods are a classic example of how this can be done analytically, though the relatively low eBird data density across the Neotropics has meant that analyses using these methods have mainly been focused on the temperate zone (Fink et al., 2020; Sullivan et al., 2009, 2014). Employing study designs that use eBird data as an adjunct to more structured surveying techniques is a possible strategy (Reich et al., 2018), as it would reduce the proportion of overall bias due to nonrandom spatial sampling in eBird while retaining the benefits of using multiple independent datasets to address the same question. As the eBird dataset is likely to be used as a basis for future species distribution modeling studies in this region that will go on to inform conservation assessments and NGO land purchasing decisions, we recommend that any such study should include a structured monitoring component, like camera trapping or acoustic monitoring, to make the conclusions of the study more relevant at specific Neotropical forest sites or narrow ecological regions.

A common question posed by research scientists in the pursuit of an efficient but effective machine learning platform is “how much training data is enough data.” Our two‐pass classification strategy demonstrated clear classification accuracy improvements over a single pass, though the degree to which our ensemble modeling strategy improved classification performance varied substantially between classes. We suspect that most of the performance improvements that could be gained beyond what we saw in our analysis would come from gathering additional survey data, iterating the data collection and training processes to increase sample sizes, and further improving the model architecture and hyperparameters. It is important to note that the main limiting factor for our use of machine learning classification has been the amount of computational power available to us, which required us to decrease the complexity of our neural networks and the resolution of our spectrograms relative to those mentioned in the literature (Kahl et al., 2019; Knight et al., 2017). While doing so allowed us to produce classification results within acceptable time constraints, this speed benefit potentially came at the cost of reduced classification accuracy. An important future goal for our analyses is to securing sufficient computational power to run the classification at full resolution to quantify improvements in accuracy, as we strongly believe that understanding the minimum acceptable resolution necessary to achieve a given level of accuracy is a crucial logistical consideration for researchers seeking to build hardware systems to support similar data processing pipelines.

One potential pitfall of using positive detections from one network to train a larger second network is that one risks missing out on gaining training examples where the acoustic profile of the call is in some way intrinsically different from the bulk of the other training examples, causing the second model to become biased toward false negatives. This could occur if the target taxa use distinct song and call vocalization types, as was indeed the case for two of the species in this study, *C. soui* and *C. variegatus*. Special effort was made to gather audio that represented the full breadth of the acoustic parameter space for each species prior to training network 1, and the relatively simple and stereotyped nature of tinamou vocalizations (with the noted caveats for *soui* and *variegatus*) alleviated some of these concerns. However, it is possible that this false‐negative bias would become more harmful for taxa with more varied vocalizations. Extreme care must be taken to ensure that training examples are fully representative of real‐world acoustic space, and research teams seeking to replicate this methodology should possess strong domain knowledge both of machine learning and of their target taxa.

Acoustic monitoring represents a promising method for studying bird biology and life history. We are particularly excited by the prospect of being able to use this SWIFT survey data in future analyses to identify the life‐history and microhabitat characteristics that result in niche partitioning in the tinamou community of lowland Madre de Dios. We anticipate that additional data collection, particularly during the wet season, and further refinement of this machine learning pipeline will allow us to build occupancy models for these species using elevation maps and vegetation structure datasets that were collected for use with the associate camera trap grid as environmental covariates (Royle & Nichols, 2003).

## CONFLICT OF INTEREST

None declared.

## AUTHOR CONTRIBUTIONS

**Reid B. Rumelt:** Conceptualization (lead); Data curation (lead); Formal analysis (lead); Funding acquisition (lead); Investigation (lead); Methodology (lead); Project administration (equal); Resources (lead); Software (lead); Supervision (equal); Writing‐original draft (equal); Writing‐review & editing (equal). **Arianna Basto:** Conceptualization (supporting); Data curation (supporting); Investigation (equal); Project administration (equal); Writing‐original draft (equal); Writing‐review & editing (equal). **Carla Mere Roncal:** Conceptualization (supporting); Investigation (supporting); Project administration (equal); Supervision (supporting); Writing‐original draft (supporting); Writing‐review & editing (equal).

## Supporting information

Table S1

Table S2

Table S3

Table S4

## Data Availability

The source audio used to create the training and validation data is publicly available from Xeno‐Canto (https://www.xeno‐canto.org) and from the Macaulay Library of Natural Sounds (https://www.macaulaylibrary.org) (catalog numbers in S2). The training and testing datasets are also archived in Data Dryad (https://doi.org/10.5061/dryad.n02v6wwxw), along with the acoustic event database collected from the survey audio.
